# New Delhi Metallo-Beta-Lactamase Inhibitors: A Systematic Scoping Review

**DOI:** 10.3390/jcm13144199

**Published:** 2024-07-18

**Authors:** Lutfun Nahar, Hideharu Hagiya, Kazuyoshi Gotoh, Md Asaduzzaman, Fumio Otsuka

**Affiliations:** 1 Department of General Medicine, Okayama University Graduate School of Medicine, Dentistry and Pharmaceutical Sciences, Okayama 700-8558, Japan; 2 Department of Infectious Diseases, Okayama University Hospital, Okayama 700-8558, Japan; 3 Department of Bacteriology, Okayama University Graduate School of Medicine, Dentistry and Pharmaceutical Sciences, Okayama 700-8558, Japanas.bmb.bd@gmail.com (M.A.)

**Keywords:** antimicrobial resistance, carbapenemase-producing *Enterobacterales*, carbapenem-resistant *Enterobacterales*, metallo-beta-lactamase, synergy, combination

## Abstract

**Background/Objectives**: Among various carbapenemases, New Delhi metallo-beta-lactamases (NDMs) are recognized as the most powerful type capable of hydrolyzing all beta-lactam antibiotics, often conferring multi-drug resistance to the microorganism. The objective of this review is to synthesize current scientific data on NDM inhibitors to facilitate the development of future therapeutics for challenging-to-treat pathogens. **Methods**: Following the Preferred Reporting Items for Systematic Reviews and Meta-Analyses (PRISMA) Extension for Scoping Reviews, we conducted a MEDLINE search for articles with relevant keywords from the beginning of 2009 to December 2022. We employed various generic terms to encompass all the literature ever published on potential NDM inhibitors. **Results**: Out of the 1760 articles identified through the database search, 91 met the eligibility criteria and were included in our analysis. The fractional inhibitory concentration index was assessed using the checkerboard assay for 47 compounds in 37 articles, which included 8 compounds already approved by the Food and Drug Administration (FDA) of the United States. Time-killing curve assays (14 studies, 25%), kinetic assays (15 studies, 40.5%), molecular investigations (25 studies, 67.6%), in vivo studies (14 studies, 37.8%), and toxicity assays (13 studies, 35.1%) were also conducted to strengthen the laboratory-level evidence of the potential inhibitors. None of them appeared to have been applied to human infections. **Conclusions**: Ongoing research efforts have identified several potential NDM inhibitors; however, there are currently no clinically applicable drugs. To address this, we must foster interdisciplinary and multifaceted collaborations by broadening our own horizons.

## 1. Introduction

Antimicrobial resistance (AMR) is a pressing global issue that requires collaborative efforts from nations and foundations worldwide [1]. Clinical and public health challenges posed by emerging AMR pathogens are particularly pronounced in low-resource settings, where enhanced laboratory capabilities and robust data collection systems are needed to fully address this health threat. Until recently, carbapenems served as last-resort treatments for Gram-negative bacterial infections [2]. However, the global emergence and rapid spread of carbapenem-resistant organisms present a significant risk of high mortality across diverse populations due to limited treatment options [3,4]. Carbapenem resistance can develop through various mechanisms, including (i) structural modifications of penicillin-binding proteins, (ii) reductions in outer-membrane porins, (iii) activation of efflux pumps, and (iv) production of β-lactamases (carbapenemases) that degrade or hydrolyze carbapenems [5]. Among these, the producibility of carbapenemases is particularly noteworthy in terms of its impact on infection prevention and treatment.

A wide range of carbapenemases are classified into Ambler Classes based on their hydrolytic profiles and catalytic substrates [6]. Class B enzymes, also known as metallo-β-lactamases (MBLs), employ zinc as a cofactor at the active site of the β-lactam ring. This class mainly includes New Delhi metallo-beta-lactamase (NDM), Verona Integron-encoded metallo-beta-lactamase (VIM), and imipenemase (IMP). Among these, NDM is the most prominent genotype capable of catalyzing a range of β-lactam antibiotics, including carbapenems, and is resistant to various β-lactamase inhibitors [7]. Since the first detection of the NDM-1 gene in *Enterobacterales* isolated from a patient traveling from India to Sweden in 2008 [8], a total of 41 NDM variants have been identified in clinically significant pathogens such as *Escherichia coli*, *Klebsiella pneumoniae*, *Acinetobacter baumannii* complex, and *Pseudomonas aeruginosa*, of which 40 variants have been deposited in the GenBank database [9,10,11]. Due to its high-level and multi-drug resistance nature, only a limited number of treatment options are available for NDM-producing bacterial infections. The endemic regions of these NDM producers have rapidly expanded worldwide, affecting communities, animals, agricultural products, and the environment [12,13], exposing an increasing number of people to untreatable infections. In the era of international travel and medical tourism, this unfavorable situation is accelerating globally [14,15].

In light of these challenges, there is significant value in promoting the development of therapeutic agents against NDM-producing bacteria. However, due to the limited research efforts in this field, progress has been modest. Nonetheless, novel antibiotics with activity against NDM producers, such as ceftazidime/avibactam plus aztreonam, aztreonam/avibactam, cefiderocol, plazomicin, and eravacycline, have recently received approval in American and European countries [16]. However, these new drugs are not yet available globally due to issues related to drug availability and cost. Combination therapy with currently available antibiotics is one approach to combat severe NDM-producing infections [16], though these strategies have not fully addressed the menace. Many studies have focused on combinatory tactics to enhance antibiotic efficacy, utilizing various compounds such as β-lactamase inhibitors, outer-membrane permeabilizers, and efflux pump inhibitors [17]. Among these, experimental and clinical investigations of combination therapy with β-lactam and β-lactamase inhibitors have been particularly explored. As a result, avibactam, relebactam, and vaborbactam have been developed and introduced to the market as serin-β-lactamase inhibitors [18,19]. However, no specific NDM inhibitors have been discovered. A recent literature review on progress in the development of MBL inhibitors summarized the molecular profiles and inhibitory mechanisms of MBLs [20]. Gu et al. have concentrated on NDM-1 inhibitors and reviewed relevant articles published after 2018, indicating chemical complexity and inconsistency [21].

Given this context, a more comprehensive evaluation of published data and a deeper discussion from a clinical applicability perspective, especially focusing on NDM inhibitors, are necessary to prepare for future crises. Therefore, our aim is to conduct a comprehensive research review of existing data on NDM inhibitors to identify promising candidates for further development.

## 2. Materials and Methods

### 2.1. Study Design and Strategy

We followed the Preferred Reporting Items for Systematic Reviews and Meta-Analyses (PRISMA) Extension for Scoping Reviews [22,23]. After a pilot search, we conducted a systematic scoping review with the following search phrases to overview MEDLINE for all peer-reviewed publications published between 1 January 2009 and 31 December 2022: “NDM inhibitor” [All Fields] OR “NDM-1 inhibitor” [All Fields] OR “NDM-1 producing bacteria” [All Fields] OR “NDM-1-producing Escherichia coli” [All Fields] OR “beta-lactamase NDM-1” [All Fields] OR “New Delhi Metallo-β-lactamase-producing *Enterobacteriaceae*” [All Fields] OR “New Delhi Metallo-β-lactamase-1” [All Fields] OR “New Delhi Metallo-β-lactamases” [All Fields] OR “MBL inhibitors” [All Fields] OR “Meropenem resistance” [All Fields] OR “In vitro Meropenem” [All Fields] OR “Overcome antibiotic resistance” [All Fields] OR “Synergistic antibacterial effects” [All Fields]. There were no language or research design filters used.

### 2.2. Eligibility Criteria

The inclusion criteria were as follows:

Peer-reviewed articles reporting results of in vitro combination tests for potential NDM inhibitors, such as checkerboard (CB) assays, time-killing assays, kinetic assays (enzyme inhibition assays using kinetic parameters such as K*i*, K*m*, K*cat*, and K*cat*/K*m* values), molecular studies, in vivo animal studies, and toxicity assays.

The exclusion criteria were as follows:

(1) Articles published in languages other than English.
(2) Conference or meeting abstracts, unrelated topics, review articles, guidelines, and commentaries.

### 2.3. Study Selection, Data Extraction, and Definition

LN and MA collected, analyzed, and assessed the selected full-text articles. Articles that met the criteria for inclusion in this study underwent a comprehensive review. We extracted information regarding the inhibiting compounds, as well as the in vitro and in vivo methods employed to confirm the combination effects and safety data from each study.

In this study, we focused on the results of the fractional inhibitory concentration (FIC) index based on the checkerboard (CB) assay to quantitatively measure the synergistic effects of the inhibitors. Generally, the FIC of an agent is calculated by dividing the minimum inhibitory concentration (MIC) of the agent when used in combination by the MIC of the agent when used alone. The FIC index is the sum of the FICs of the combined drugs. Interactions between the combined drugs were quantified using the FIC index as follows: an FIC index of ≤0.5 was defined as synergistic, and an FIC index of ≥0.5 to ≤4.0 was considered indifferent [24].

The time-killing curve assay is also a fundamental approach to confirm the synergistic efficacy of two or more agents. In this study, we defined a bactericidal effect as a bacterial volume reduction of 3 log_10_ CFU/mL or more at any time during incubation when the drugs were combined. Conversely, bacteriostatic activity was characterized by a reduction of less than 3 log_10_ CFU/mL compared to the initial inoculum.

### 2.4. Data Synthesis and Statistical Analysis

Data processing and aggregation were performed using Microsoft Excel^®^ software version 2021 (Microsoft Corporation, Redmond, WA, USA). We did not perform any statistical analysis since this is a descriptive study.

## 3. Results

### 3.1. Search Results and Study Selection

**Figure 1:**
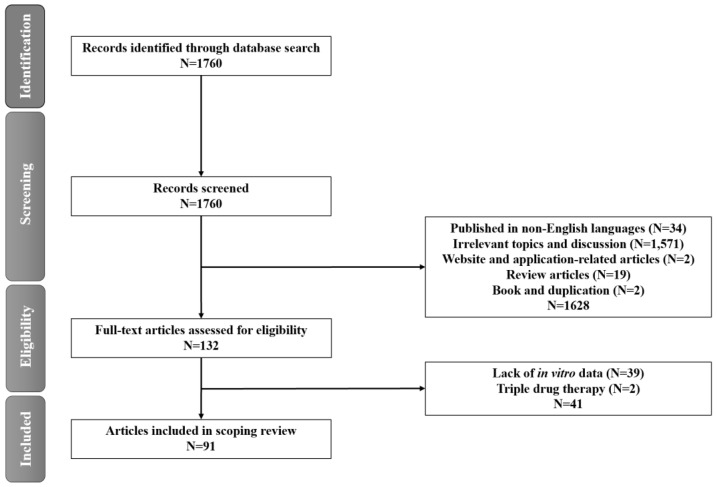
Flowchart of the study process.

The flowchart depicting the stages of article collection is presented in Figure 1, illustrating the process of identifying relevant reports, screening records, evaluating eligibility, and making final determinations for inclusion or exclusion in accordance with the PRISMA flow diagram. The initial search of MEDLINE databases yielded 1760 articles, which underwent further eligibility screening, resulting in the exclusion of 1628 articles. Subsequently, 132 full-text articles were assessed, and 39 articles lacking experimental data and 2 articles related to triplet agent therapy were excluded. Ultimately, 91 articles (comprising 89 original articles and 2 letter-type articles) were selected for the review. 

### 3.2. Description of the Review Results

**Figure 2:**
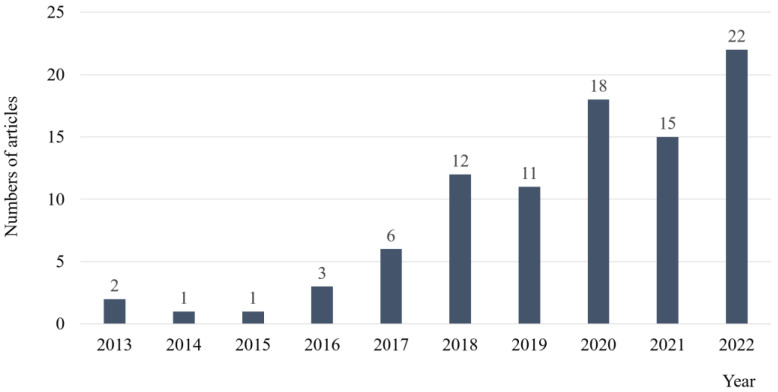
Annual numbers of eligible articles, by publication year.

The number of articles has significantly increased, especially in the last five years: 12 in 2018, 11 in 2019, 18 in 2020, 15 in 2021, and 22 in 2022 (Figure 2). A summary of 91 articles reporting 154 potential NDM inhibitors is provided in Table 1 [25,26,27,28,29,30,31,32,33,34,35,36,37,38,39,40,41,42,43,44,45,46,47,48,49,50,51,52,53,54,55,56,57,58,59,60,61,62,63,64,65,66,67,68,69,70,71,72,73,74,75,76,77,78,79,80,81,82,83,84,85,86,87,88,89,90,91,92,93,94,95,96,97,98,99,100,101,102,103,104,105,106,107,108,109,110,111,112,113,114,115]. All 91 studies were found to have conducted CB assays. Time-killing curve assays, kinetic assays, molecular investigations, in vivo (animal- or cell-based) combination studies, and toxicity assays were carried out in 26 (28.6%), 41 (45.1%), 66 (72.5%), 30 (33.0%), and 44 (48.4%) of the studies, respectively. Various strains of NDM-producing bacteria were used in both in vitro and in vivo studies (Supplementary Table S1). The two most common isolates employed were *Escherichia coli* and *Klebsiella pneumoniae*, followed by other E*nterobacterales* species, *Pseudomonas aeruginosa*, and *Acinetobacter baumanii*. Clinical, recombinant, standard, reference, and wild strains were used in 57 (62.6%), 27 (29.7%), 25 (27.5%), 3 (3.3%), and 2 (2.2%) of the studies, respectively, including some duplications.

Out of the 154 NDM inhibitors extracted from 91 eligible articles, we specifically identified 47 potential inhibitors in 37 articles, where the FIC index was determined based on the CB assay (Table 2). Among these, eight compounds had already received approval from the United States FDA. Almost all of these compounds exhibited synergistic effects with an FIC index of less than 0.5. However, some cases of indifferent results were identified when various bacterial strains were tested. Out of these, 14 (37.8%) studies included data on the time-killing curve assay. Bacteriostatic effects were reported in 4 studies, while 10 studies (involving 11 inhibitors) demonstrated bactericidal effects.

Additionally, 12 studies (32.4%) conducted kinetic assays, in which kinetic parameters were calculated. Molecular investigations were conducted in 23 (62.2%) studies, with molecular docking and molecular dynamic simulations being commonly employed (15 out of 25 studies, 60%). To validate the efficacy of combination therapy, 10 studies (27%) presented in vivo animal data, all of which used mouse models. To assess the safety of the inhibitory drugs used, 13 (35.1%) studies reported results of toxicity assays using in vivo models. Notably, none of the compounds exhibited apparent toxic effects.

## 4. Discussion

In this scoping review, we have compiled the presently available data on NDM inhibitors published in MEDLINE. Among the various experimental methods used to evaluate the efficacy of drug combinations, we specifically focused on the FIC index calculated through the CB assay, which serves as a fundamental approach to determine the synergistic effects of two distinct drugs. Since 2014, a total of 47 compounds have been investigated as potential NDM inhibitors, with 8 of them having received approval from the United States FDA. These FDA-approved drugs include various substances such as methimazole, withaferin A, fisetin, isoliquiritin, cefmetazole, pterostilbene, cefoxitin, and tetracycline [34,37,38,74,78,81,83]. In addition to the CB assay, bactericidal effects were observed in 10 compounds through time-killing curve assays, of which 4 substances (methimazole, fisetin, isoliquiritin, and cefmetazole) had already received FDA endorsement [34,38,74,78]. No further investigations had been conducted for methimazole and cefmetazole [34,78], whereas the effectiveness and safety of combining fisetin or isoliquiritin were additionally confirmed through other approaches [38,74]. Regrettably, there were no inhibiting agents that seemed readily available for clinical use, and none of these are within the reach of clinicians.

Kinetic assays and molecular investigations represent more advanced methods for ascertaining combination efficacy. Comparing molecular affinities among compounds of interest using kinetic parameters such as K*i*, K*m*, K*cat*, and K*cat*/K*m* can provide insights into inhibitory activity from an enzymatic perspective. Molecular docking simulations of potential inhibitors are well-established computational methods for analyzing molecular binding modes. Among these two elaborated approaches, molecular docking and molecular dynamic simulations were more frequently performed (62.2% vs. 32.4%). Eleven studies did not conduct either of these methods [25,33,34,55,69,74,76,96,97,106,108], while nine studies evaluated both [38,46,48,49,75,80,88,95,113]. Additionally, in vivo animal studies were performed in 10 studies [38,46,48,75,80,81,82,95,96,113], suggesting that the tested compounds, including H2dpa derivatives, sulfamoylfuran-3-carboxylic acid derivatives, peptidomimetic 4, pterostilbene, and aspergillomarasmine A, may hold promise as inhibitors.

For unapproved compounds, ensuring their safety is essential for potential future clinical use. In this sense, toxicity assays provide particularly important data. In our review, 13 out of 37 studies (35.1%) conducted these assays, primarily using a mouse model. Notably, no inhibitors with apparent toxicity were reported. However, it is essential to mention that zinc-chelating agents may not be suitable for therapeutic use due to their well-documented toxicity to human cells [25,44,49,56,82,88].

Our study has a few limitations that should be acknowledged. First, we conducted our search exclusively on MEDLINE due to the unavailability of access to other databases. This could potentially lead to an underestimation of relevant articles. In fact, our search approach failed to include boron-based inhibitors, such as taniborbactam, xeruborbactam, and zidebactam, which have the potential to be available in clinical settings. Possibilities of reporting bias should also be considered. Second, we only included articles in the English language, which may restrict comprehensiveness and affect generalizability. Third, our search period was up to the end of December 2022, which should have been extended to the time of drafting, because an increasing number of relevant articles have been reported in the literature. Due to time constraints, we could not afford to do so. Fourth, the presence of publication bias should be taken into consideration. Data that could be unfavorable for the inhibitors might not have been included in the articles. Fifth, clinical strains may possess various antimicrobial resistance mechanisms, and, therefore, the combination of NDM inhibitors may not necessarily exhibit synergistic effects in clinical settings. Finally, the assessment of the quality of the included studies was not fully performed, although it is a crucial aspect of the review study to ensure the validity and reliability of the conclusion.

## 5. Conclusions

In summary, there are currently no NDM inhibitors available for therapeutic use. While previous efforts have borne fruit in identifying some potential compounds, there is still a long road ahead to discover clinically applicable and outstanding NDM inhibitors. Just as the development of serine-β-lactamase inhibitors has set an example, it is time for NDM inhibitor research to follow suit. For this purpose, the establishment of a laboratory and clinical research platform under interdisciplinary collaborations is necessary. We believe that our review work will contribute to advancing this challenging journey.

## Figures and Tables

**Table 1:** A summary of 91 articles reporting the 154 potential NDM inhibitors.

No.	Year	NDM Inhibitors	CB Assay	TKC Assay	Kinetic Assay	Molecular Methods	In Vivo Study	Toxicity Assay	Ref.
1	2022	EDTA, Captopril, Ciprofloxacin	○	×	×	×	×	×	[25]
2	2022	1,2,4-triazole-3 thiones derivative	○	×	○	○	×	○	[26]
3	2022	1,2-Isoselenazol-3(2H) derivatives	○	×	×	○	×	○	[27]
4	2022	Ebselen scaffold	○	○	×	○	×	×	[28]
5	2022	Cephalosporin-Tripodalamin conjugate	○	○	×	○	○	○	[29]
6	2022	Fragment-based compounds	○	×	○	○	×	×	[30]
7	2022	Adapelen	○	○	×	○	×	×	[31]
8	2022	Aromatic Schiff bases	○	×	×	○	×	○	[32]
9	2022	Bismuth dichloride	○	×	×	×	×	○	[33]
10	2022	Alpha Lipoic acid, methimazole	○	○	×	×	×	×	[34]
11	2022	QDP-1 (Phenyl ring)	○	×	○	○	×	×	[35]
12	2022	Trans-cephalosporin	○	×	○	○	×	○	[36]
13	2022	Withaferin A	○	×	×	○	×	×	[37]
14	2022	Fisetin	○	○	○	○	○	×	[38]
15	2022	Quinolinyl-Sulphonamides sulphonyl esters	○	×	○	○	○	○	[39]
16	2022	Emerione A, Asperfunolone A	○	×	×	○	○	×	[40]
17	2022	Risedronate, Methotrexate	○	×	○	○	×	×	[41]
18	2022	Aspergillomarasmine A analogue	○	×	×	○	○	×	[42]
19	2022	Unithiole derivative	○	×	○	○	○	×	[43]
20	2022	Nitroxoline derivative	○	○	×	○	×	○	[44]
21	2022	Indole-2-carboxylates derivative	○	×	×	○	○	○	[45]
22	2022	Di-thiocarbamates-copper	○	○	○	○	○	○	[46]
23	2021	Alkylthio-substituted thiols derivatives	○	×	×	○	×	×	[47]
24	2021	H2dpa derivatives	○	○	○	○	○	○	[48]
25	2021	Thiosemicarbazone derivative	○	○	○	○	×	○	[49]
26	2021	Thiosemicarbazones derivative	○	×	○	○	×	×	[50]
27	2021	N-acylhydrazones derivative	○	×	○	○	○	○	[51]
28	2021	Azetidinimines derivatives	○	×	○	○	○	○	[52]
29	2021	N-Sulfamoylpyrrole-2-carboxylates derivatives	○	×	×	×	○	×	[53]
30	2021	Indole-carboxylate derivative	○	×	×	○	×	×	[54]
31	2021	Cephalosporin-prodrug	○	×	×	×	×	×	[55]
32	2021	Benzimidazole and benzoxazole zinc chelator	○	×	×	○	×	×	[56]
33	2021	Diaryl-substituted thiosemicarbazone derivative	○	○	○	×	○	○	[57]
34	2021	Fragment-based compound	○	○	×	×	○	○	[58]
35	2021	2-Mercaptomethyl-thiazolidines derivative	○	×	○	×	×	○	[59]
36	2021	Thiosemicarbazone derivatives	○	○	○	○	○	○	[60]
37	2021	D-captopril’s derivatives	○	×	×	○	×	×	[61]
38	2020	4-Amino-1,2,4-triazole-3-thione-derived Schiff bases	○	×	○	○	○	○	[62]
39	2020	Carnosic acid	○	○	×	○	×	×	[63]
40	2020	Chemical peptide sequences	○	×	×	○	×	○	[64]
41	2020	Disulfiram, nitroxoline, 5-amino-8-hydroxyquinoline, DOTA, cyclam, TPEN	○	○	○	×	○	○	[65]
42	2020	ANT2681 (thiazolyl acid derivatives)	○	×	○	○	○	○	[66]
43	2020	H2dedpa derivatives	○	○	×	○	×	○	[67]
44	2020	1,2-benzisothiazol-3(2H) derivative	○	×	×	○	×	○	[68]
45	2020	Carboxylates small molecules	○	×	×	×	×	×	[69]
46	2020	Metal complex scaffold (PDTC2-Fe)	○	×	○	○	×	×	[70]
47	2020	ZINC05683641	○	×	×	○	×	×	[71]
48	2020	PcephPT (cephalosporin prochelator)	○	×	○	○	×	×	[72]
49	2020	α-hydrazono carboxylic acid fragments	○	×	×	○	×	×	[73]
50	2020	Isoliquiritin	○	○	×	×	×	×	[74]
51	2020	Sulfamoyl hetero-arylcarboxylic acids derivatives	○	×	○	○	○	○	[75]
52	2020	Amino-carboxylic acid analogues	○	×	×	×	×	×	[76]
53	2020	Disulfiram	○	○	○	○	×	×	[77]
54	2020	Cefmetazole	○	○	○	×	×	×	[78]
55	2020	3-bromopyruvate	○	×	○	○	○	○	[79]
56	2019	Peptidomimetic 4 (PEP4)	○	○	○	○	○	○	[80]
57	2019	Pterostilbene	○	○	×	○	○	×	[81]
58	2019	Mercapto propionamide derivatives	○	×	×	○	○	○	[82]
59	2019	Cefoxitin, tetracycline	○	×	○	×	×	×	[83]
60	2019	Silver nanoparticles (AgNPs)	○	×	×	×	×	○	[84]
61	2019	H_2_-dedpa derivative	○	○	○	○	×	○	[85]
62	2019	Tris-(2-picolyl) amine	○	○	×	○	×	×	[86]
63	2019	Ebsulfur scaffolds	○	×	×	○	○	○	[87]
64	2019	1,4,7-Triazacyclononane	○	○	○	○	×	○	[88]
65	2019	Azolyl-thio acetamides derivatives	○	×	×	○	×	○	[89]
66	2019	Tannic acid	○	×	×	○	×	○	[90]
67	2018	Dipicolinic acid derivative	○	×	○	○	×	○	[91]
68	2018	Magnolol	○	○	×	○	×	×	[92]
69	2018	Di-thiocarbamate derivatives	○	×	×	×	×	○	[93]
70	2018	Tris-picolylamine-based zinc chelators	○	×	○	×	○	○	[94]
71	2018	1,2-benzisoselenazol-3(2H) derivatives	○	×	○	○	○	○	[95]
72	2018	Dipicolyl-vancomycin conjugate	○	×	×	×	○	○	[96]
73	2018	Crude soy saponins	○	×	×	×	×	×	[97]
74	2018	Small carboxylic acid derivatives	○	×	○	○	×	×	[98]
75	2018	Thiol based inhibitors	○	×	×	○	×	○	[99]
76	2018	Fragment-based derivative	○	×	×	○	×	×	[100]
77	2018	Embelin	○	×	×	○	×	×	[101]
78	2018	Dithiocarbamate derivatives	○	○	○	×	×	○	[102]
79	2017	Triazol-thiol derivatives	○	×	○	×	×	×	[103]
80	2017	Peptide-conjugated phosphorodiamidate morpholino oligomer (PPMO)	○	×	×	×	○	×	[104]
81	2017	2-mercapto-3-phenylpropionic acid derivative	○	×	×	○	×	×	[105]
82	2017	Aspergillomarasmine A derivative	○	×	×	×	○	×	[106]
83	2017	AW01120, BTB02323	○	×	○	○	×	○	[107]
84	2017	Hibiscus cannabinus, Tamarindus indica, Combretum albidum, Hibiscus acetosella, Hibiscus furcatus, Punica granatum	○	×	×	×	×	×	[108]
85	2016	Captopril Stereoisomers	○	×	×	○	×	×	[109]
86	2016	Metal chelators (1) DPA, (2) TPEN	○	×	×	×	×	○	[110]
87	2016	Bisthiazolidines (compound-f L-CS319)	○	○	○	○	×	○	[111]
88	2015	Ebselen	○	×	○	○	×	×	[112]
89	2014	Aspergillomarasmine A	○	×	○	○	○	×	[113]
90	2013	Polyketide compounds	○	×	×	○	×	×	[114]
91	2013	Thiophene-carboxylic acid derivatives	○	×	○	○	×	×	[115]

CB, checkerboard; TKC, time-killing curve; ○ indicates a conducted assay, while × indicates that assay was not performed. Various assays were adopted for each compound.

**Table 2:** Detailed summary of 37 articles reporting the 47 NDM inhibitors with data for the fractional inhibitory concentration (FIC) index.

No.	Year	Tested Compounds [Combined Drugs] ^(1)^	** FIC Index by CB Assay	TKC Assay	Kinetic Assay	Molecular Investigation ^(2)^	In Vivo Study (Animal)	*** Toxicity Assay (Model)	Ref.
1	2022	(1) EDTA (2) Captopril (3) Ciprofloxacin [MEPM, IPM]	(1) Synergistic (2) Synergistic and indifferent (3) Synergistic and indifferent	-	-	-	-	-	[25]
2	2022	1, 2-Isoselenazol-3(2H) derivatives [MEPM]	Synergistic	-	-	MDS	-	Not toxic (mammalian cell)	[27]
3	2022	Adapelen [MEPM]	Synergistic and indifferent	Bacteriostatic	-	MDS	-	-	[31]
4	2022	Bismuth dichloride (C4) [MEPM]	Synergistic	-	-	-	-	Toxic (human embryonic kidney cell)	[33]
5	2022	(1) Alpha Lipoic acid (2) Methimazole * [MEPM]	All synergistic	*Bactericidal*	-	-	-	-	[34]
6	2022	Withaferin A * [IPM]	Synergistic	-	-	MDS	-	-	[37]
7	2022	Fisetin * [MEPM]	Synergistic and indifferent	*Bactericidal*	Performed	MDS	Mouse	-	[38]
8	2022	(1) Emerione A, (2) Asperfunolone A [MEPM, IPM, CTRX, ABPC]	-	-	-	MDS	-	-	[40]
9	2022	Nitroxoline derivative [IPM]	Synergistic	*Bactericidal*	-	SAR	-	Non-specific ^(3)^ (endothelial cell)	[44]
10	2022	Di-thiocarbamates-copper (SA09-Cu) [MEPM]	Synergistic	Bacteriostatic	Performed	SAR	Mouse	Less toxic (mouse)	[46]
11	2021	H2dpa derivatives	All Synergistic	*Bactericidal*	Performed	MDS	Mouse	Less toxic (mouse)	[48]
12	2021	Thiosemicarbazone derivative [MEPM]	Synergistic	Bacteriostatic	Performed	MDS	-	-	[49]
13	2021	Indole-carboxylate derivative [MEPM]	Synergistic	-	-	ITC	-	-	[54]
14	2021	Cephalosporin-prodrug [MEPM]	Synergistic	-	-	-	-	-	[55]
15	2020	1,2-benzisothiazol-3(2H) derivative [MEPM]	Synergistic	-	-	MDS, ESI-MS	-	Acceptable toxicity (human embryonic kidney cell)	[68]
16	2020	Carboxylates small molecules [MEPM]	Synergistic and indifferent	-	-	-	-	-	[69]
17	2020	ZINC05683641 [MEPM]	Synergistic	-	-	MDS	-	-	[71]
18	2020	Isoliquiritin * [MEPM]	Synergistic and indifferent	*Bactericidal*	-	-	-	-	[74]
19	2020	Sulfamoyl hetero-arylcarboxylic acid derivatives [MEPM]	All synergistic	-	Performed	Protein Crystallization	Mouse	Less toxic (mouse)	[75]
20	2020	Aminocarboxylic acid analogues [MEPM]	All synergistic	-	-	-	-	-	[76]
21	2020	Cefmetazole * [MEPM]	Synergistic	*Bactericidal*	Performed	-	-	-	[78]
22	2019	Peptidomimetic 4 (PEP4) [MEPM]	Synergistic and indifferent	*Bactericidal*	Performed	MDS	Mouse	Non-specific ^(3)^ (mammalian cell)	[80]
23	2019	Pterostilbene * [MEPM]	Synergistic and indifferent	Bacteriostatic	-	MDS	Mouse	-	[81]
24	2019	Mercapto propionamide derivative [MEPM]	All synergistic	-	-	X-ray crystallography	Mouse	Non-specific ^(3)^ (mouse)	[82]
25	2019	(1) Cefoxitin * (2) Tetracycline * [DRPM]	All Synergistic	-	Performed		-	-	[83]
26	2019	Tris-(2-picolyl) amine (TPA) [MEPM]	Synergistic	*Bactericidal*	-	MDS	-	-	[86]
27	2019	1,4,7-Triazacyclononane [MEPM]	Synergistic	*Bactericidal*	Performed	MDS	-	Non-specific ^(3)^ (immortalized liver carcinoma cells)	[88]
28	2018	Magnolol [MEPM]	Synergistic	*Bactericidal*	-	MDS	-	-	[92]
29	2018	1,2-benzisoselenazol-3(2H) derivatives [MEPM]	Synergistic and indifferent	-	Performed	ESI-MS	Mouse	Less toxic (larvae)	[95]
30	2018	Vancomycin analogue (dipicolyl-vancomycin conjugate) [MEPM]	Synergistic	-	-	-	Mouse	Non-specific ^(3)^ (mouse model, mammalian cell)	[96]
31	2018	Crude soy saponins [PIPC, ABPC, MPIPC, PCG]	Synergistic	-	-	-	-	-	[97]
32	2018	Embelin [IPM]	Synergistic	-	-	MDS	-	-	[101]
33	2017	Triazol-thiol derivatives [CTX, MEPM]	All synergistic	-	Performed	-	-	-	[103]
34	2017	2- mercapto-3-phenylpropionic acid derivative [MEPM]	Synergistic	-	-	ITC	-	-	[105]
35	2017	Aspergillomarasmine A derivatives [MEPM]	All synergistic	-	-	-	-	-	[106]
36	2017	(1) Hibiscus cannabinus (2) Tamarindus indica (3) Combretum albidum (4) Hibiscus acetosella (5) Hibiscus furcatus (6) Punica granatum [MEPM]	All synergistic	-	-	-	-	-	[108]
37	2014	Aspergillomarasmine A [MEPM]	Synergistic	-	Performed	ICP-MS	Mouse	-	[113]

CB, checkerboard; TKC, time-killing curve. ^(1)^ Abbreviations of combined drugs: MEPM, meropenem; IPM, imipenem; CTRX, ceftriaxone; ABPC, ampicillin; DRPM, doripenem; PIPC, piperacillin; MPIPC, oxacillin; PCG, benzylpenicillin; CTX, cefotaxime. ^(2)^ Abbreviations of methods: MDS, molecular docking and molecular dynamic simulation; SAR, structural activity relationship analysis; ESI-MS, electrospray ionization mass spectrometry; ITC, isothermal titration assay; ICP-MS, inductively coupled mass spectrometry. ^(3)^ Non-lethal doses were used. * FDA-approved drug. ** Synergistic effect was determined as that with an FIC index of ≤0.5. *** “Not toxic” was defined as those without any side effects shown in the experimental model. “Less toxic” was defined as when any signs of drug-associated adverse effects were observed.

## Data Availability

Available with a valid reason from a corresponding author.

## Supplementary Materials

The following supporting information can be downloaded at: https://www.mdpi.com/article/10.3390/jcm13144199/s1, Table S1: Species and types of NDM-producing bacteria used in each study [116].
